# Supplementary material for: The interactome of intact mitochondria by cross-linking mass spectrometry provides evidence for coexisting respiratory supercomplexes
Source: Mol Cell Proteomics. 2017 Dec 8;17(2):216–32. doi: 10.1074/mcp.RA117.000470 (PMC5795388; doi:10.1074/mcp.RA117.000470)
Supplement: Supplemental Data [file supp_RA117.000470_133922_0_supp_23978_fzffwf.zip › spectra_annotation/mito_DR_spectra_annotation/498-1-4-1-14-1.pdf]

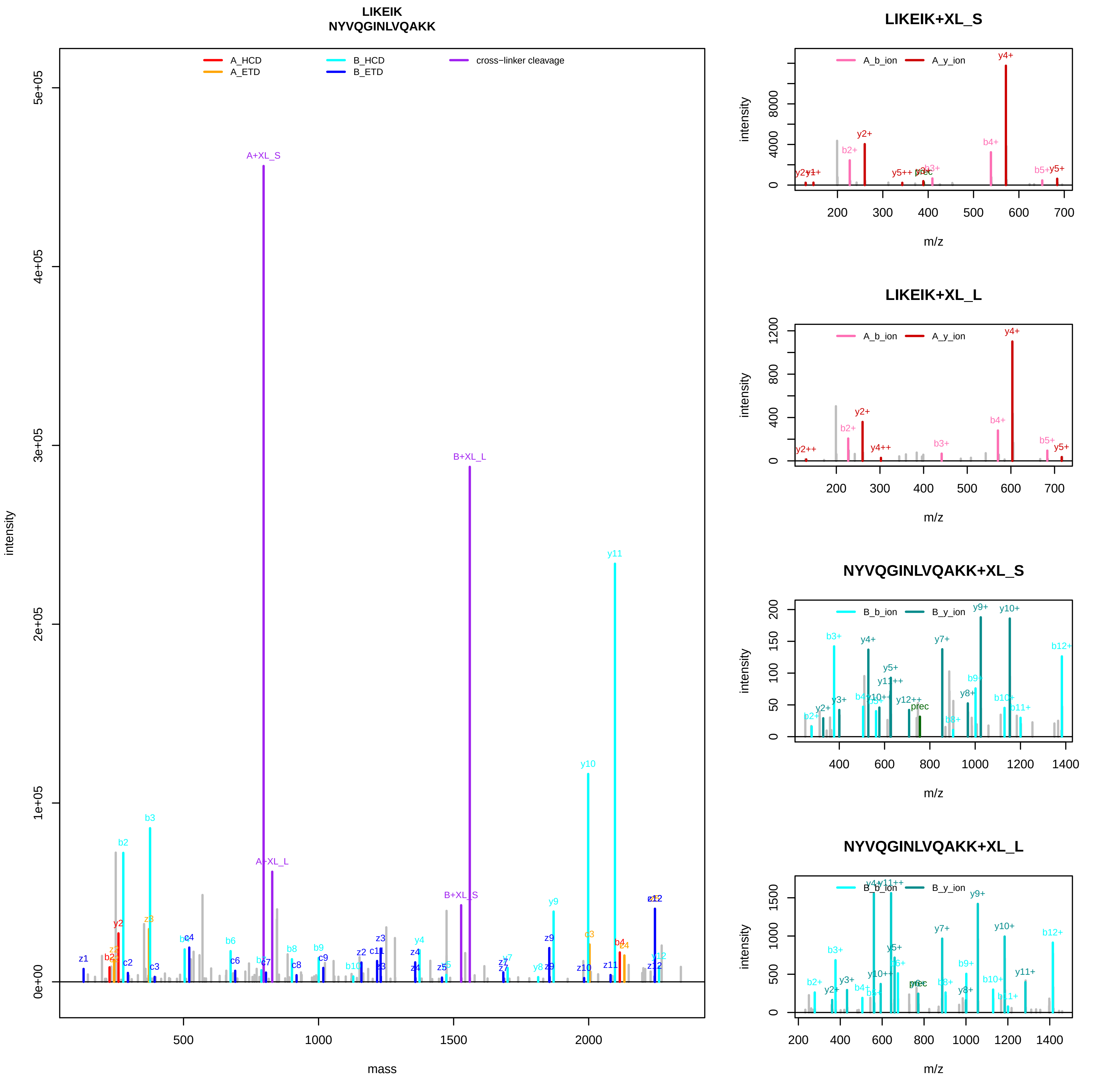

LIKEIK
NYVQGINLVQAKK
A_HCD
A_ETD
B_HCD
B_ETD
cross−linker cleavage
A+XL_S
B+XL_L
A+XL_L
B+XL_S
y11
y10
y9
b3
b2
y2
z3
z12
intensity
mass
0e+00
1e+05
2e+05
3e+05
4e+05
5e+05
500
1000
1500
2000
LIKEIK+XL_S
A_b_ion
A_y_ion
y4+
y2+
b2+
b4+
b5+
y5+
prec
m/z
0
4000
8000
200
300
400
500
600
700
LIKEIK+XL_L
y2++
y4++
b3+
0
400
800
1200
NYVQGINLVQAKK+XL_S
B_b_ion
B_y_ion
y9+
y10+
b3+
y4+
y7+
y5+
y11++
b12+
b9+
y8+
b10+
0
50
100
150
200
400
600
800
1000
1200
1400
NYVQGINLVQAKK+XL_L
y4+y11++
y7+
y10+
b12+
y11+
0
500
1000
1500
